# Functional Cooperativity between ABCG4 and ABCG1 Isoforms

**DOI:** 10.1371/journal.pone.0156516

**Published:** 2016-05-26

**Authors:** Zoltán Hegyi, László Homolya

**Affiliations:** Institute of Enzymology, Research Centre for Natural Sciences, Hungarian Academy of Sciences, Budapest, Hungary; University of Cambridge, UNITED KINGDOM

## Abstract

ABCG4 belongs to the ABCG subfamily, the members of which are half transporters composed of a single transmembrane and a single nucleotide-binding domain. ABCG proteins have a reverse domain topology as compared to other mammalian ABC transporters, and have to form functional dimers, since the catalytic sites for ATP binding and hydrolysis, as well as the transmembrane domains are composed of distinct parts of the monomers. Here we demonstrate that ABCG4 can form homodimers, but also heterodimers with its closest relative, ABCG1. Both the full-length and the short isoforms of ABCG1 can dimerize with ABCG4, whereas the ABCG2 multidrug transporter is unable to form a heterodimer with ABCG4. We also show that contrary to that reported in some previous studies, ABCG4 is predominantly localized to the plasma membrane. While both ABCG1 and ABCG4 have been suggested to be involved in lipid transport or regulation, in accordance with our previous results regarding the long version of ABCG1, here we document that the expression of both the short isoform of ABCG1 as well as ABCG4 induce apoptosis in various cell types. This apoptotic effect, as a functional read-out, allowed us to demonstrate that the dimerization between these half transporters is not only a physical interaction but functional cooperativity. Given that ABCG4 is predominantly expressed in microglial-like cells and endothelial cells in the brain, our finding of ABCG4-induced apoptosis may implicate a new role for this protein in the clearance mechanisms within the central nervous system.

## Introduction

The ABCG1 and ABCG4 proteins belong to the G subfamily of ATP binding cassette (ABC) transporters. Unlike most other eukaryotic ABC transporters, these proteins consist of only one nucleotide binding domain (NBD) and one transmembrane domain (TMD), therefore, are called ABC half-transporters. Another characteristic feature of the members of the G subfamily is the reverse domain order, meaning that unlike in most ABC transporters, the NBD is localized at the N-terminus of the proteins.

In full-length ABC transporters the two NBDs cooperatively form the ATP binding sites, therefore, it is commonly accepted that ABC half-transporters have to dimerize to generate a functioning unit. The ABCG2 transporter has been demonstrated to function as a homodimer or a homomultimer in various systems [1–4]. In contrast, the closely related ABCG5 and ABCG8 proteins have been shown to function as obligate heterodimers [5–7]. Homodimerization of ABCG1 has also been suggested previously by co-immunoprecipitation, non-reducing SDS-PAGE, and cross-linking [8–10].

Since ABCG1 and ABCG4 share 72% overall amino acid identity, heterodimerization of these proteins has also been predicted [11]. This hypothesis is further supported by the observations that their *Drosophila* ortholog, the *White* gene product is known to form heterodimers [12]. The dissimilar tissue distribution of ABCG1 and ABCG4, however, contradicts the implication of heterodimerization. Merely neuronal tissues, eye, and induced macrophages are the overlapping regions where both ABCG1 and ABCG4 were detected simultaneously [13–18]. Our previous observation that the ATPase activity of ABCG1 expressed in Sf9 cells was inhibited by the inactive mutant variant of ABCG4 provided further support for heterodimer formation [19].

Two major splice variants of the mammalian ABCG1, which differ in a 12 amino acid-long segment, have been described [20]. In human monocyte-derived macrophages and Thp-1 cells both isoforms are expressed, however, the short variant is the predominant form [20, 21]. The murine ABCG1 lacking the 12 amino acid-long insert, corresponds to short form of human ABCG1 [22].

Regarding their function, ABCG1 and ABCG4 have been suggested to play a role in cellular lipid/sterol regulation. ABCG1 has been shown to facilitate cholesterol efflux from cells to HDL particles, and been proposed to participate in generation of HDL particles in concert with ABCA1 [9, 14, 20, 23]. It should, however, be noted that cholesterol was released form ABCG1-expressing cells to LDL, PC vesicles, PC/ApoAI discs, BSA, and cyclodextrin as effectively as to HDL [9, 14, 20, 23]. We previously demonstrated that the functional ABCG1 induces apoptosis in macrophages and other cell types, providing an alternative explanation for non-specific cholesterol efflux from ABCG1-expressing cells, and suggesting an unconventional role for ABCG1 [24]. Both the short and long variants of the human ABCG1 have been shown to promote cholesterol efflux from cells [21], however, the apoptotic effect of ABCG1 was only demonstrated by using the long variant [24]. The function of ABCG4 is even more enigmatic. Due to the high sequence similarity to ABCG1 and its transcriptional regulation by LXR, ABCG4 has also been proposed to participate in the lipid/sterol regulation in the tissues and cell types where expressed, i.e., macrophages and various neural cells [13, 14, 25]. However, studies in ABCG4 knock out mice did not verify this suggestion, since LXR-induced cholesterol efflux from macrophages was independent of ABCG4 expression [23]. More recently, Murphy at al. demonstrated that ABCG4 is expressed in megakaryocyte progenitors, and contributes to the regulation of platelet production via a mechanism, which involves HDL- and ABCG4-dependent cholesterol efflux from this cell type [26].

In the present study, we investigated the dimerization properties of ABCG4, and its interactions with the ABCG1 variants. Based on the homology, high level sequence similarity, and capability of heterodimerization, we hypothesized similar functions for ABCG1 and ABCG4, and investigated the apoptotic effects of the various dimers. Using inactive mutant forms and assessing a functional readout allowed us to demonstrate functional cooperativity between ABCG1 and ABCG4.

## Materials and Methods

### Expression of ABCG proteins in mammalian cells and growth curve analysis

The cDNA of the full-length isoform of ABCG1 (NP_004906.3) and ABCG4 (NP_001135977.1) were cloned into pEGFP-N1 vector, leaving a stop codon and frame shift between EGFP and the encoded protein sequences. The shorter (NP_058198.2), 1998 nucleic acid long isoform of ABCG1 was generated by site-directed mutagenesis by using GGCCCTCTGAAGAGGACTCCTCGTCCATGG and CCATGGACGAGGAGTCCTCTTCAGAGGGCC primer pair. The cDNAs of ABCG1 isoforms were sequenced to confirm the identity with the Pubmed database. The cDNA of ABCG2 (NP_004818.2) was cloned into the pcDNA3.1 vector. To generate ABCG1, ABCG4 and ABCG2 variants tagged N-terminally with GFP the sequences of the transporters were cloned into pEGFP-C1vector as describe previously [27]. The HEK293 cells (ATCC) were maintained in complete DMEM in humidified CO_2_ incubator at 37°C. For transfection, 4×10^5^, 8×10^4^, or 4×10^4^ cells were seeded into 6-, 24-well plates (Greiner), or 8-well (Nunc) Lab-Tek II chambers, respectively. After 24h, the transfection was performed by using Fugene HD (Roche) reagent according to the manufacturer’s instruction. Transfection media were replaced after 8 hours, and the protein expression was verified by immunoblot or immunofluorescence staining at different time points after transfection.

Growth curve analyses were performed on 24-well plates for a 3-day period following transfection. Total living cell number was counted at 24, 48, and 72 hours with trypan blue counterstaining. To investigate the change in the expression levels of ABCG proteins in the transfected cultures, all the cells in the entire well were harvested 24, 48, 72 and 96 hours after transfection and subjected to Western blot analysis (see more details below). To generate cell lines stably expressing ABCG4 or ABCG4_K108M_, 0.5 mg/ml G418 (Gibco) was added to the culturing medium 48 hours after transfection, the cells were subsequently maintained in this selection medium changed on a regular basis. The protein expression levels were determined after an 18-day selection period by Western blotting. The MDCKII cell lines (ATCC) stably expressing the myc-tagged ABCG5 and HA-tagged ABCG8 proteins were generated by retroviral transduction.

### Immunoblot experiments

ABCG4-specific monoclonal antibody was developed similarly as described previously for antibody against ABCG1 [24]. Briefly, GST fusion protein with the nucleotide binding domain of ABCG4 was produced, and used for immunization. Sera were screened for ABCG4-positivity and ABCG1-negativity. Selected mice were sacrificed and hybridoma cells were generated, cloned and retested. Monoclonal antibody was purified from supernatant of the best clone, and its isotype was determined, i.e, IgG2a.

Western blot analysis of whole cell lysates were performed as described previously [28] with some modifications. Briefly, the cells were suspended in 1x Laemmli buffer and sonicated three times for 10 seconds at 4°C. Samples, containing 40 μg protein or in some indicated cases the entire cell lysate, were loaded onto a Laemmli-type 7.5% SDS-polyacrylamide gel and separated. Electroblotting of the proteins onto PVDF membrane was carried out in Tris buffer containing high concentration of glycine and 4% methanol at 200 mA for 1.5 hour. Immunodetection was performed by using specific monoclonal antibodies; clone 6G1/7 for ABCG1, clone 19C11 for ABCG4, BXP-21 (Abcam) for ABCG2, anti-HA (Covance Babco) for epitope-tagged ABCG8 and anti-myc (Roche) for myc-tagged ABCG5. All antibodies were diluted 500-fold in TBS-Tween-milk buffer. HRP-conjugated goat anti-mouse IgG secondary antibody (Jackson Immunoresearch, 1:10.000), and enhanced chemiluminescence technique was applied to detect specific bands. For loading control, a mouse anti-Na^+^K^+^ ATPase antibody (Biomol, 1:1600) was used, with the exception when the entire cells lysates were subjected to Western blot analysis.

### Coimmunoprecipitations

24 hour after transfection, HEK293 cells were scraped from the 6 well plate in lysis buffer (50 mM Tris pH 7.4, 150 mM NaCl, 0.5% NP-40) supplemented with Roche protease inhibitor cocktail and 1 mM PMSF. Samples were then sonicated for 10 seconds in an ultrasonicator. After sonication, whole cell lysates were centrifuged for 10 min at 4°C at 17000g. 1/10 of the supernatants were precipitated by 4.5% TCA and resolved in Laemmli buffer. 10 μg of appropriate antibody (anti-GFP, anti-ABCG1 or anti-ABCG4) had been coupled to 40 μl of protein G-sepharose slurry for 2 hours at RT and the 9/10 of the supernatant was added and immunoprecipitated for 2 hours at 4°C on a microtube shaker. Beads were washed three times in PBS containing 0.5% NP-40 and boiled in 2x Laemmli buffer to elute immunoprecipitated proteins. Samples were analyzed by Western blot technique as described above.

### Immunofluorescence staining

Immunofluorescence staining was carried out as described previously [24] with minor modifications. Briefly, transiently transfected HEK293 cells were washed in Dulbecco’s modified PBS, fixed and permeabilized with 4% paraformaldehyde and pre-chilled methanol. Following fixation, the cells were incubated for 1 hour at RT in blocking buffer (0.2% BSA, 1% fish gelatin, 0.1% TX-100, and 5% goat serum). Samples were then labeled for 1 hour at RT in blocking buffer containing specific primary antibody. Anti-pan-cadherin, anti-giantin, and anti-calnexin antibodies purchased from Abcam were diluted in blocking buffer in 1:100, 1:500, and 1:100 dilutions, respectively. After a gentle wash, the cells were incubated with species- or isotype-specific, AlexaFluor-conjugated secondary antibodies (Life Technologies/Thermo Fisher Scientific) diluted in blocking buffer (1:250). Green (505–525 nm) and far red (>660 nm) fluorescence images of the immunostained samples were acquired at 488 and 633 nm excitations, respectively. An Olympus FV500-IX confocal laser scanning microscope with PLAPO 60× (1.4) oil immersion objective were used for imaging.

### Detection of apoptotic cells

For quantitative assessment of apoptosis, the cells were incubated in Annexin V binding buffer containing 20-fold diluted Alexa Fluor 488-labeled Annexin V (Life Technologies/Thermo Fisher Scientific) and 5 μM Hoechst33342 (Life Technologies/Thermo Fisher Scientific) for 3 min. At least 3 separate fields of view were acquired for each condition by confocal microscopy using an UPLAPO 40× (0.85) dry objective. Annexin V positive cells and total cell number were determined on the basis of green and blue fluorescence, respectively. The results were expressed as percentage of total cell number. For co-staining of apoptotic cells and ABCG4, the cells were first subjected to Alexa Fluor 647- conjugated Annexin V (Life Technologies/Thermo Fisher Scientific) (20-fold dilution, 3 min), then fixed and immunostained as described above. To detect caspase-3 activity and Annexin V positivity in same experiment, the cells were incubated with 10 μM PhiPhiLuxG2D2 (Calbiochem) and 10% FCS for 20 min, washed twice, then subjected to Alexa Fluor 488-labeled Annexin V in Annexin V binding buffer for 3 min. Results are expressed as mean ± S.E.M. For statistical analysis, Student's t-test was used to evaluate significant differences. (p < 0.01) in comparison with controls.

## Results

### Dimer formation of ABCG4

**Fig 1:**
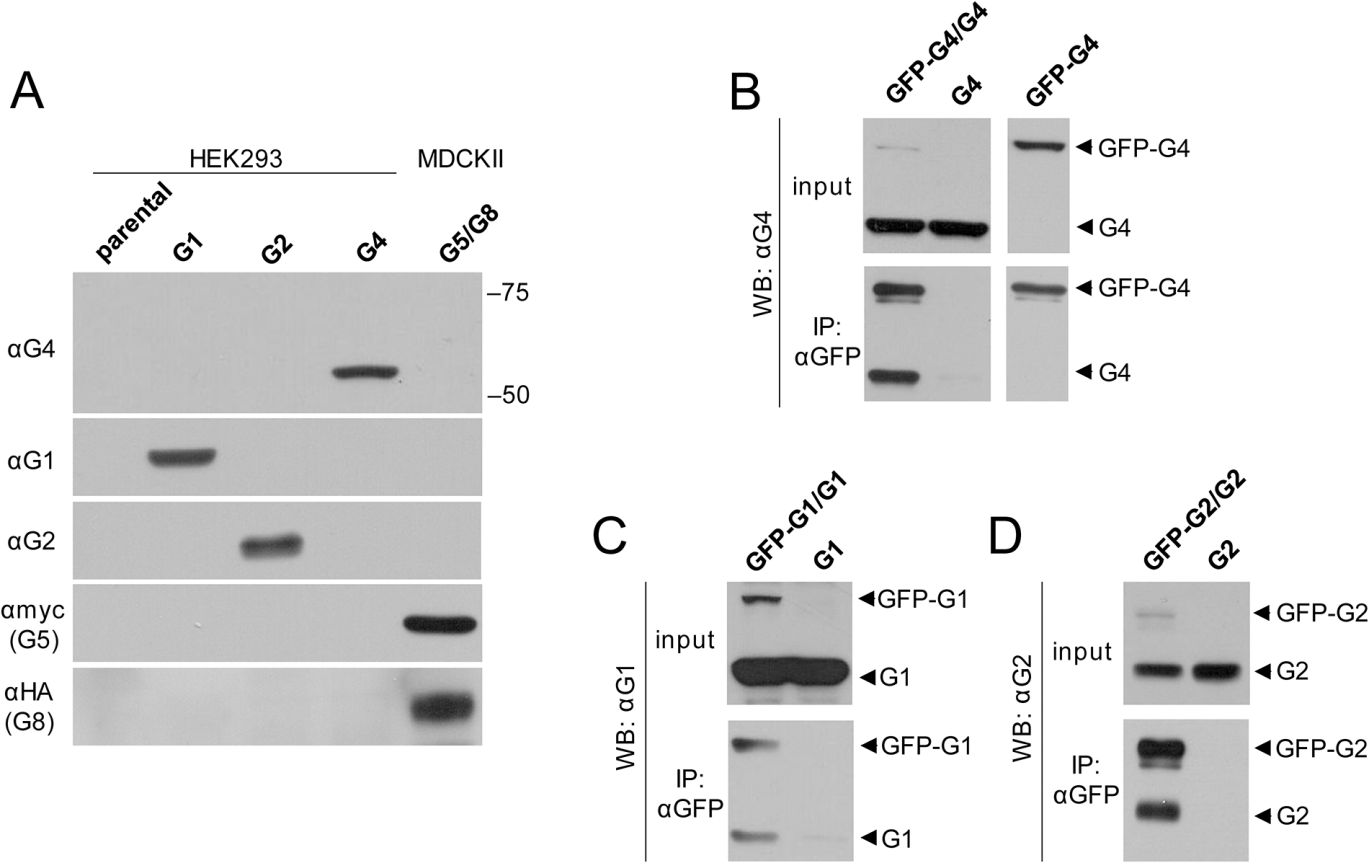
Homodimer formation of ABCG4, ABCG1 and ABCG2. (A) We have generated specific antibodies against ABCG1 (αG1) and ABCG4 (αG4). The specificity of the applied antibodies was confirmed by Western Blot technique performed with 40 μg of whole cell lysates from HEK cells expressing ABCG1 (G1), ABCG2 (G2), ABCG4 (G4), or myc-tagged ABCG5 along with HA-tagged ABCG8 (G5/G8). Parental HEK cells were used as a negative control. (B) Co-immunoprecipitation experiments were performed with HEK cells co-transfected with GFP-ABCG4 (GFP-G4) and ABCG4 (G4). Single transfected samples served as controls. Cells were lysed and an anti-GFP antibody was used for immunoprecipitation. Western blots of precipitates were developed by our anti-ABCG4 (αG4) antibody. The protein expressions were also verified (input). The presence of both untagged and GFP-tagged ABCG4 in the precipitate of co-transfected cells indicates dimer formation. Similar experiments were performed with GFP-ABCG1 (GFP-G1) and ABCG1 (G1) (C), or GFP-ABCG2 (GFP-G2) and ABCG2 (G2) pairs (D). Like ABCG2, both ABCG1 and ABCG4 can form homodimers.

To detect ABCG4 expression, we generated a monoclonal antibody against ABCG4 with an approach that we previously used to raise an antibody against ABCG1, utilizing the GST-fused N-terminal domain of the ABC protein for immunization [24]. The specificity of the monoclonal antibody was confirmed by Western blot analysis with whole cell lysates from HEK293 cells transfected with ABCG1, ABCG2, or ABCG4, as well as from MDCKII cells transduced with c-myc-tagged ABCG5 and HA-tagged ABCG8 (Fig 1A). For negative controls, cell lysates from the parental cell line was used. The anti-ABCG4 antibody recognized a single band at the expected molecular weight (app. 60 kDa) in the ABCG4-containing sample, and did not cross-react with any other ABCG proteins. Noteworthy, even ABCG1 was not recognized by the anti-ABCG4 antibody, despite the 72% amino acid identity of these proteins.

To explore whether the ABCG4 protein is glycosylated, ABCG4-expressing cells were treated with tunicamycin (5 μg/ml, 24 h). Since ABCG2 is known to be glycosylated, ABCG2-expressing cells were used for positive control. In HEK cells, ABCG2 is fully glycosylated as demonstrated in our previous study [27] and also by the appearance of a single band in Fig 1A. We found that tunicamycin treatment altered the apparent molecular weight of ABCG2, but had no effect on the migration of ABCG4 (data not shown), implying that ABCG4 is not glycosylated. This finding is in agreement with previous results using a tagged variant of ABCG4 and glycosydase digestion to study ABCG4 glycosylation [20].

Our previous studies on the ATPase activity of ABCG1 implicated that ABCG1 is capable of forming homodimers and also heterodimers with ABCG4 [19]. To study whether ABCG4 forms a homodimer, we co-expressed GFP-tagged ABCG4 with the untagged protein in HEK293 cells, and immunoprecipitated the proteins with anti-GFP antibody. In these experiments, the untagged version was expressed in excess to increase the efficacy. Western blot of the precipitate developed with our anti-ABCG4 antibody clearly demonstrates the dimer formation (Fig 1B). For negative control, a precipitate from cells expressing ABCG4 alone was used. To prove that the lower band is not a degradation product of GFP-ABCG4, similar experiment was performed with cells expressing GFP-ABCG4 alone (Fig 1B). In control experiments, homodimer formation of ABCG1 and ABCG2 was also demonstrated by using the same co-immunoprecipitation approach (Fig 1C and 1D).

**Fig 2:**
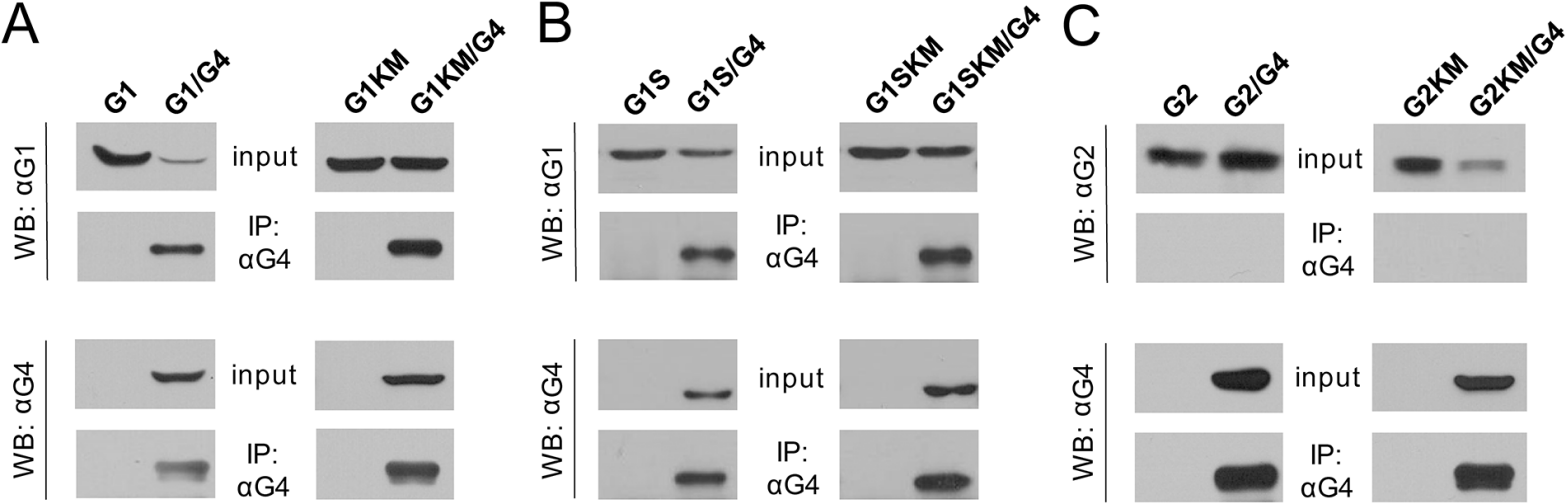
ABCG4 forms heterodimer with either isoform of ABCG1. (A) The full-length isoform of ABCG1 (G1) or its inactive mutant variant (G1KM) was co-expressed with ABCG4 (G4) in HEK293 cells. Cells were lysed, the protein expression was verified (input) and co-immunoprecipitation was performed using our anti-ABCG4 (αG4) antibody. Both wt ABCG1 and its inactive mutant were co-immunoprecipitated with ABCG4, indicating heterodimer formation between ABCG1 and ABCG4. (B) Similar results were obtained with cells co-expressing the short isoform of ABCG1 (G1S) or its non-functional mutant version (G1SKM). (C) In contrast, when cells were co-transfected with ABCG4 and wild type or the inactive variant of ABCG2 (G2 or G2KM), only ABCG4 was detected in the precipitate, demonstrating that no heterodimer formation occurs between ABCG2 and ABCG4.

To examine whether ABCG4 forms heterodimer with ABCG1, the two proteins were co-expressed in HEK293 cells and immunoprecipitated with the anti-ABCG4 monoclonal antibody. From the two isoforms of ABCG1, first the long variant was investigated. Detection of both ABCG1 and ABCG4 in the precipitate suggests heterodimer formation (Fig 2A). For negative control, cells expressing ABCG1 alone were used, and ABCG1 was not detected in the precipitate, demonstrating the specificity of the co-immunoprecipitation experiments. Since in subsequent experiments we used an inactive ABCG1 variant, ABCG1_K124M_, which contains a missense mutation in the ATP-binding site of the protein, we also investigated whether this mutant can also form heterodimer with ABCG4. As demonstrated in Fig 2A, the inactive ABCG1 variant was also co-immunoprecipitated with ABCG4, demonstrating that the heterodimer formation is not affected by the mutation. The inverse of this experiment, that is immunoprecipitation with anti-ABCG1 antibody followed by development with anti-ABCG4 antibody, also verified the heterodimer formation (S1 Fig).

To investigate whether the 12 amino acid long insert has a role in the dimerization, we performed similar co-immunoprecipitation experiments using the short isoform of ABCG1 (ABCG1S) and its inactive form. We found that these variants also co-immunoprecipitated with ABCG4 (Fig 2B). In control experiments, ABCG2 and its inactive form, ABCG2_K86M_ were co-expressed with ABCG4 in HEK293 cells. As demonstrated in Fig 2C, none of the ABCG2 variants associated with ABCG4. Our results indicate specific physical interaction of ABCG4 with both the full length and the short variant of ABCG1.

### Subcellular localization of ABCG4

**Fig 3:**
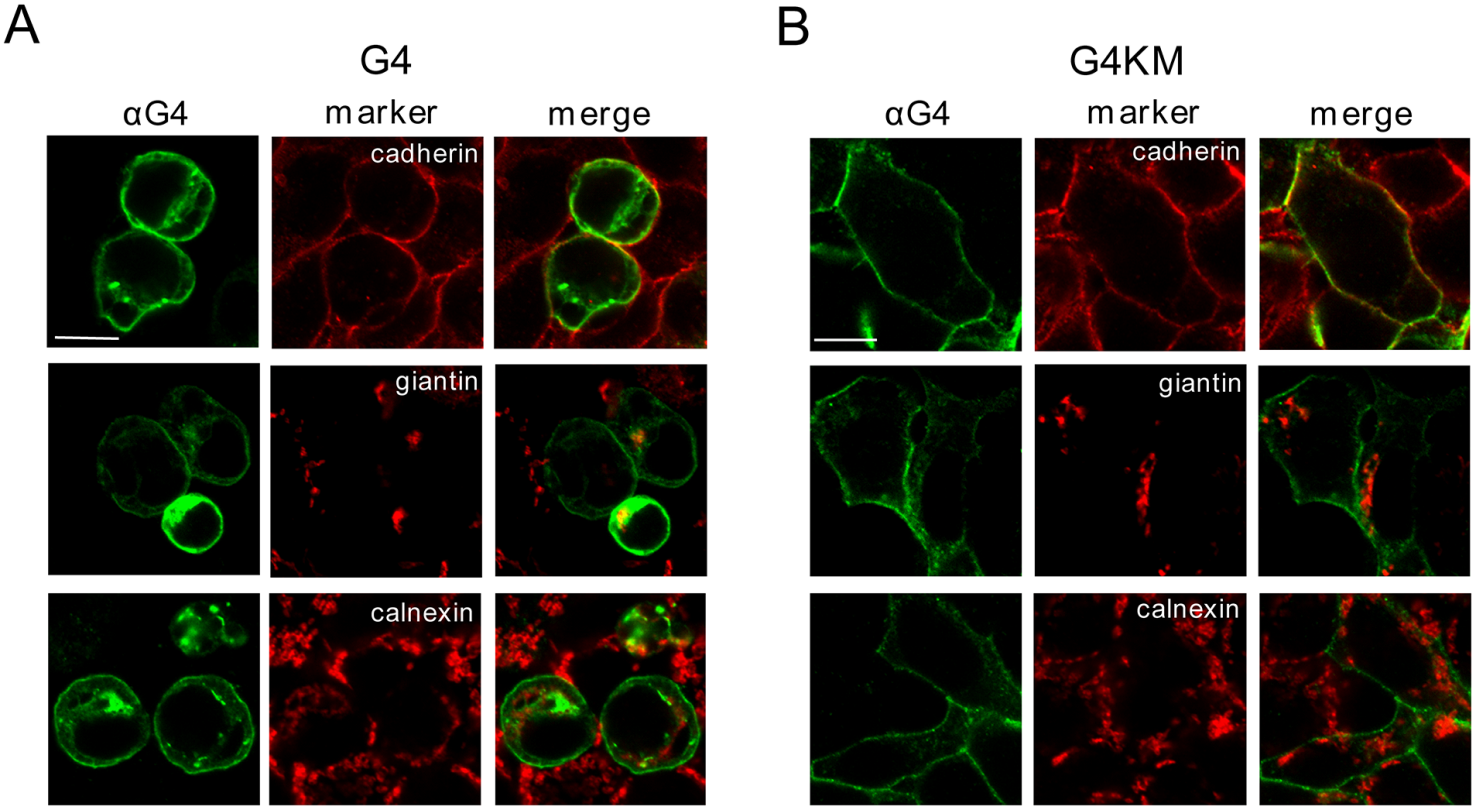
Subcellular localization of ABCG4. The wild type (G4) or the inactive mutant form (G4KM) of ABCG4 was expressed in HEK293 cells, immunostained with anti-ABCG4 antibody (αG4) and visualized by confocal microscopy. The subcellular compartments were identified by using specific markers for the plasma membrane (cadherin), the Golgi complex (giantin), and the endoplasmic reticulum (calnexin). (A) The wild type ABCG4 protein was predominantly localized to the plasma membrane, however, some intracellular staining was also observed, which was co-localized with the Golgi marker. (B) The mutant form (G4KM) exhibited even more pronounced plasma membrane localization. Scale bars—10 μm.

Despite the increasing number of publications on ABCG4, only a few studies examined the subcellular localization of this protein, even though there is no consensus in this regard. To explore this issue, we transfected HEK293 cells with ABCG4, and immunostained them with our anti-ABCG4 antibody. We found that ABCG4 was predominantly localized to the plasma membrane, which was reinforced by ABCG4 colocalization with cadherin, a plasma membrane marker (Fig 3A). Some additional intracellular staining was observed, which was overlapping with the immunostaining of the Golgi apparatus-resident protein, giantin. In contrast, the endoplasmic reticulum marker, calnexin showed distinct subcellular localization from that of the transporter. Since ABCG4 is normally expressed in neural tissues, we also expressed this protein in cell lines of neural origin, such as N2a and SH-SY5Y, and observed similar subcellular distribution to that seen in HEK293 cells (data not shown).

We also studied the localization of the inactive mutant form of ABCG4_K108M_, and found that this variant exhibited similar subcellular distribution (Fig 3B). A slight difference was that the plasma membrane localization of the mutant form was even more pronounced. Markedly different was, however, the morphology of the cells transfected with the wild type or the inactive ABCG4. This finding is in accordance with our previous observations with cells expressing ABCG1 [24]. The morphological changes, such as rounding up and detachment, seen in cells expressing the wt ABCG4 are characteristic of apoptotic cells. These features were not observed in ABCG4_K108M_-expressing cells (Fig 3B). N2a and SH-SY5Y cells transfected with the wild type or inactive mutant ABCG4 exhibited similar morphological characteristics to that seen in HEK293 cells (data not shown).

### Growth rate and expression levels in ABCG4-transfected cells

**Fig 4:**
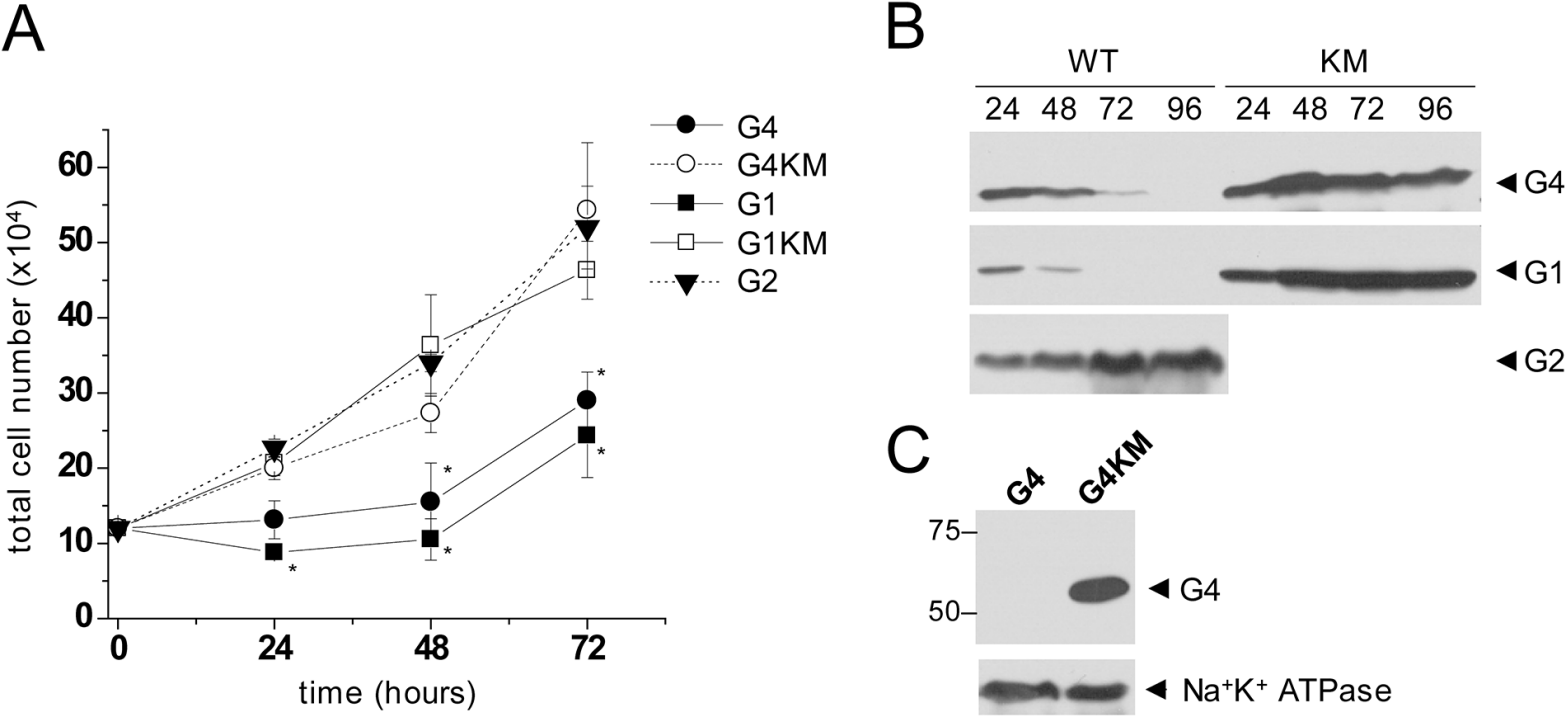
Growth rate and expression levels in ABCG1- and ABCG4-transfected cells. (A) HEK293 cells were transiently transfected with ABCG4 (G4), ABCG1 (G1), ABCG2 (G2), or with catalytic site mutant variants of ABCG4 and ABCG1 (G4KM, G1KM). The cell growth of these cultures was monitored for 72 hours. Cells expressing the wild type ABCG4 or ABCG1 protein showed inhibited growth capacity as compared to cells expressing the inactive mutants or the wild type ABCG2 (G2). (B) The expression level of ABCG proteins was also assessed by Western analysis. The total expression of the transgene rapidly declined in the cultures transfected with the wild type ABCG4 or ABCG1, whereas stable expression levels were observed in cells expressing the inactive mutants or the wild type ABCG2. Numbers at the top indicate the elapsed time in hours. (C) Following a three week long selection with G418, no ABCG4 expression was detected in cultures transfected with wild type protein (G4), whereas expression of the inactive mutant (G4KM) persisted. For loading control Na^+^K^+^ ATPase was used.

Our previous observation of ABCG1-induced apoptosis [24] and the morphological changes seen in wt ABCG4-expressing cells implies that ABCG4 may also provoke apoptosis. To test this hypothesis, first we monitored the cell growth in HEK293 cells transfected with wt ABCG4 or its inactive mutant form, ABCG4_K108M_. For comparison, cells expressing wt ABCG1, ABCG1_K124M_, and wt ABCG2 were used. Cells transfected with either wt ABCG4 or wt ABCG1 exhibited significantly smaller growth rate than cells expressing their inactive mutant counterparts, or wt ABCG2 (Fig 4A). It is noteworthy that wt ABCG1-expressing cells showed slightly slower growth kinetics than the wt ABCG4-transfected cultures. To investigate the temporal change in the expression levels of the various ABCG protein variants in the entire cultures, in parallel experiments, all the cells were collected from the culturing wells at different time points and subjected to Western blot analysis. We found that the protein levels of wt ABCG4 and wt ABCG1 rapidly declined, whereas the expression of ABCG4_K108M_, ABCG1_K124M_, and wt ABCG2 persisted (Fig 4B). In accordance with this observation, the expression of wt ABCG4 was lost, when we attempted to generate an ABCG4-expressing stable cell line by three week long continuous selection with G418 (0.5 mg/ml). In contrast, a cell line stably expressing ABCG4_K108M_ was successfully established by the same procedure (Fig 4C). Taken together, the expression of functional (wt) ABCG4 or ABCG1 attenuates cell growth, and represents a selective disadvantage in the cell cultures.

### Functional expression of ABCG4 induces apoptosis

**Fig 5:**
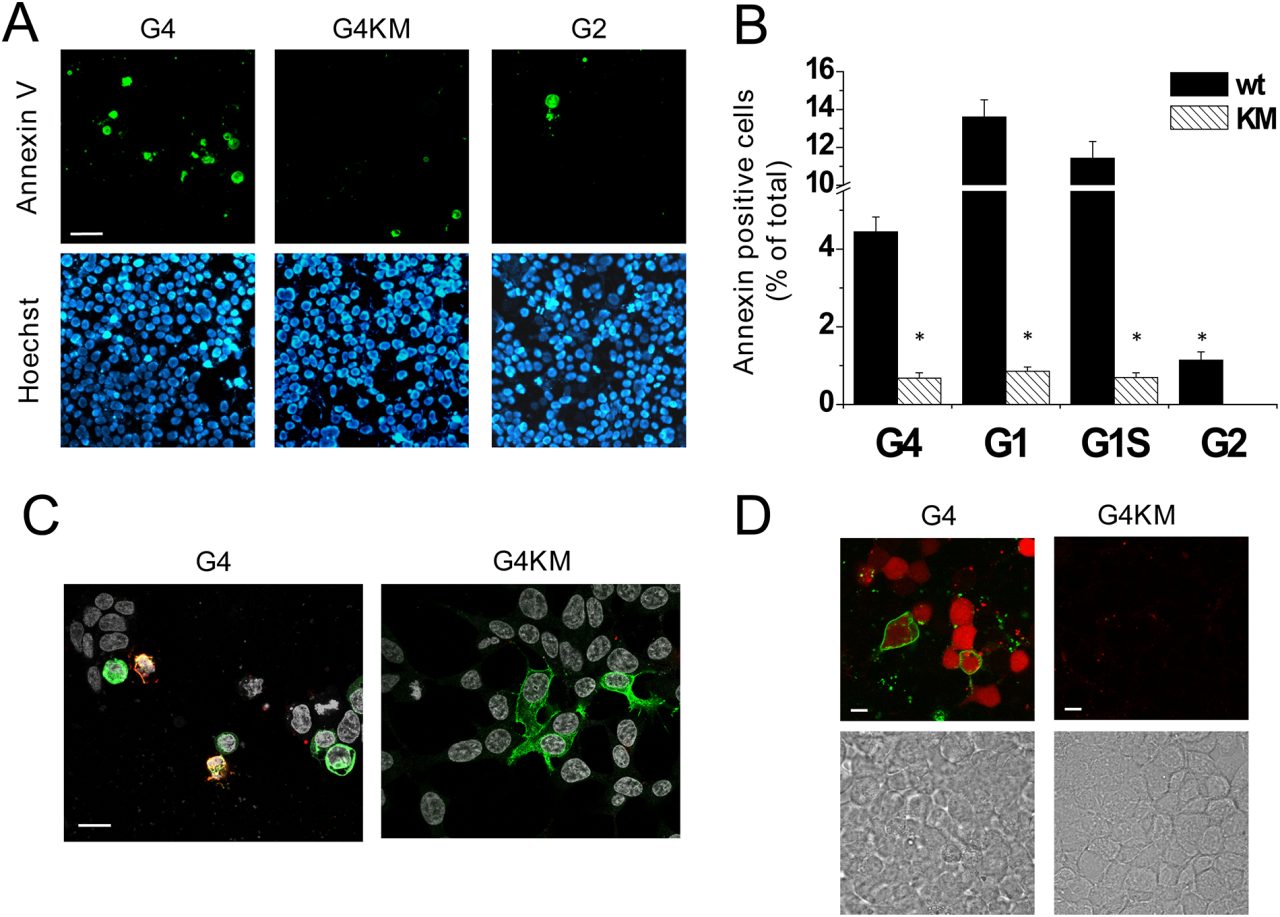
ABCG4 induces apoptosis. (A) HEK293 cells were transfected with wild type (wt) ABCG4 or its inactive mutant (G4 or G4KM, respectively). ABCG2-expressing cells served as a negative control (G2). Apoptotic cells in cultures were visualized by fluorescently labeled Annexin V (green). Lower panels depict Hoechst 33342 nuclear staining (blue) in the same fields of view. Similar experiments with the ABCG1 isoforms and their inactive mutant variants are shown in S3 Fig. (B) The quantitative results are expressed as the percentage of apoptotic cells of total cells. The mean values ± S.E.M. obtained from at least 3 independent transfections are shown. The fraction of apoptotic cells was significantly larger in cultures transfected with the wt ABCG4 or ABCG1 than in cultures expressing the inactive mutants (KM), or the wild type ABCG2. Asterisks indicate significant differences as compared to wild type expressing cells (p < 0.01). (C) Co-staining of ABCG4 expression (green) and Annexin V binding (red) in cultures transfected with wt G4 or G4KM demonstrates the connection between the functional expression of ABCG4 and apoptosis. (D) Parallel detection of Annexin V binding (green) and caspase-3 activity (red) in cell cultures transfected with wt or inactive mutant ABCG4 confirmed that the functional ABCG4-induced apoptosis. DIC images on the lower panels demonstrate the presence of cell in the same fields of view.

To explore whether ABCG4 expression leads to apoptosis similarly to that seen in ABCG1-expressing cells, we studied phosphatidyl serine (PS) externalization, an early apoptotic event, in cells transfected with the wild type or the inactive mutant form of ABCG4. In these experiments, we used fluorescently labeled Annexin V, which specifically binds to PS on the cell surface. To quantify the fraction of apoptotic cells in the cultures, 24 hours after transfection we counted the Annexin V-positive cells, and the total cell number was determined by nuclear staining with Hoechst 33342 (Fig 5A). For comparison, similar experiments were performed with cell cultures transfected with ABCG2. PS translocation was initiated by the expression of functional ABCG4, whereas hardly any apoptotic cells were found in cultures transfected with either ABCG4_K108M_ or wt ABCG2. 24 hours after transfection, when the Annexin V-binding assay was carried out, the expression levels of wt ABCG4 and ABCG4_K108M_ were comparable, as demonstrated in Fig 4B.

The fraction of apoptotic cells in cultures transfected with wt ABCG4 was monitored over time. The number of apoptotic cells gradually increased until 24 hours after transfection, subsequently a decline was observed (S2A Fig). The ABCG4 expression, determined by Western blot analysis, showed similar kinetics in the transient transfection system (S2B and S2C Fig). Dose-response relationship demonstrates a close correlation between ABCG4 expression and apoptosis (S2D Fig).

In our previous studies, we demonstrated the apoptotic effect of ABCG1, using longer isoform of the transporter [24]. To investigate whether the short variant of ABCG1, which is the predominant form of ABCG1 in humans, also induce apoptosis in cells, we measured Annexin V binding in cells transfected with both isoforms of ABCG1, as well as with the corresponding inactive mutant variants. The expression of short isoform of ABCG1 also resulted in high number of apoptotic cells in the cell culture, similar to that seen in cultures transfected with the full-length transporter (S3 Fig). Fig 5B depicts the quantitative evaluation of Annexin V-binding experiments, suggesting that ABCG4 and both isoforms of ABCG1 induce apoptosis, and this effect depends on the activity of these transporters. In contrast, the expression of the active form of ABCG2, which is closely related to ABCG1 and ABCG4, has no significant apoptotic effect.

The ABCG4-induced apoptosis was verified by using additional approaches, i.e., co-staining of apoptotic cells and ABCG4 expression, as well as assaying the activation of caspase-3 protease in the ABCG4- transfected cultures. As demonstrated in Fig 5C, Annexin V binding is concomitant with the expression of the functional ABCG4, but not with that of the inactive mutant variant. In addition, a large number of cells with high caspase-3 activity was detected in cultures transfected with the wt ABCG4, whereas no caspase-3 activation was seen in cells transfected with ABCG4_K108M_, further supporting that the activity of the transporter is required for induction of apoptosis (Fig 5D).

### Functional interaction between ABCG4 and ABCG1

**Fig 6:**
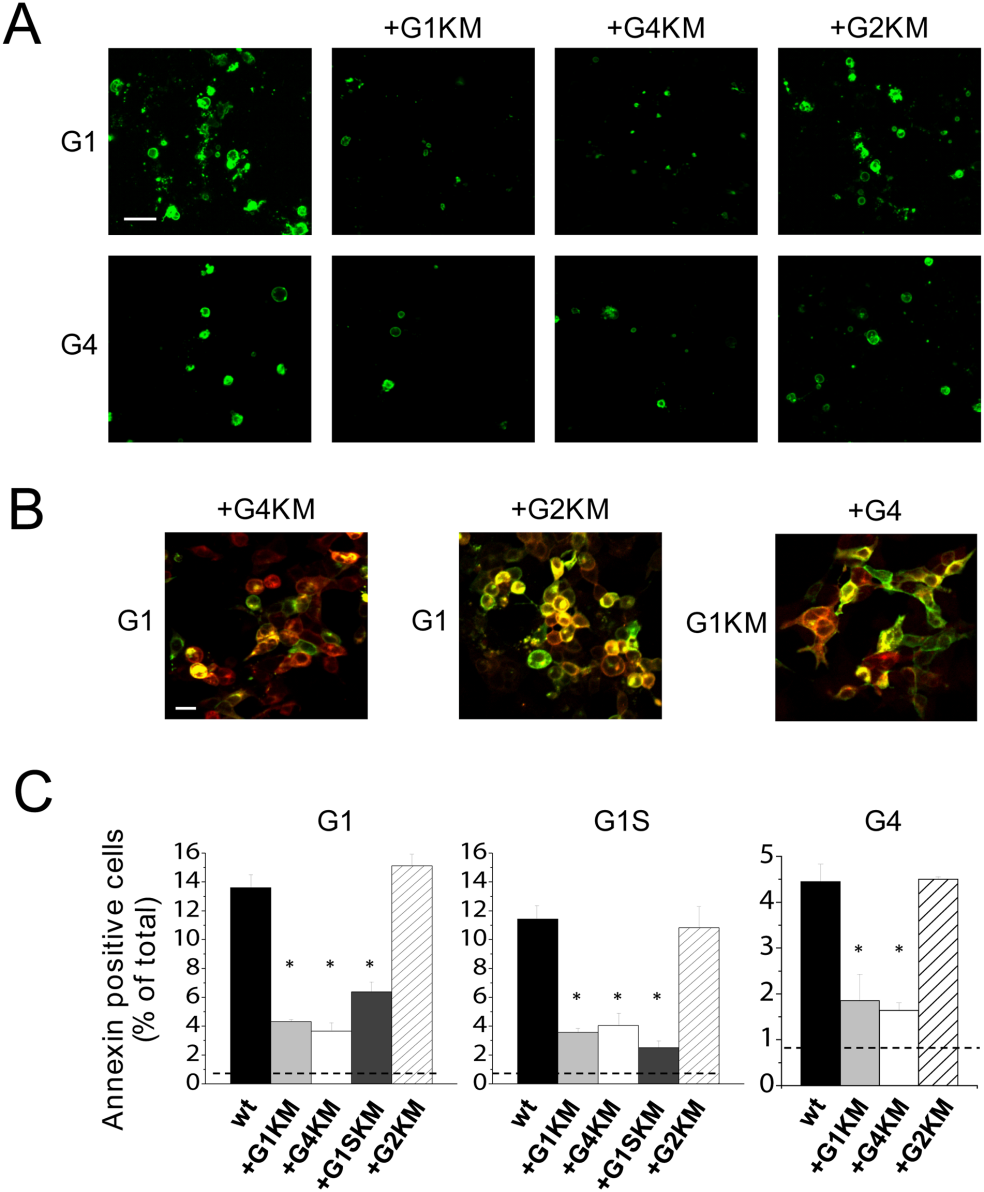
Functional interaction between ABCG4 and ABCG1. (A) To test the functional interaction between ABCG proteins, the wild type ABCG1 (G1, upper panels) or the wild type ABCG4 (G4, lower panels) was co-expressed with inactive mutant variants of the studied ABCG proteins (G1KM, G4KM, or G2KM) as indicated at the top. The apoptotic cells were identified by Annexin V binding (green). (B) The co-expression of the proteins was confirmed by dual immunostaining wherever isotype difference of the specific antibodies allowed. ABCG1 expression is shown in green, whereas the partners indicated at the top are depicted in red. (C) Quantitative evaluation of Annexin V binding experiments revealed that the fraction of the apoptotic cells were significantly reduced by the co-expression with the inactive mutant variant of ABCG1 (either isoform) or ABCG4, but not with that of ABCG2, as compared to the cultures expressing wild type (wt) ABCG1 alone. Similar results were obtained with the wild type short isoform of ABCG1, or with wt ABCG4. Asterisks represent significant differences as compared to the cultures expressing the wild type protein alone (p < 0.01).

In the experiments shown in Figs 1 and 2, we demonstrated that ABCG1 and ABCG4 can form both homodimers and heterodimers with one another. The co-immunoprecipitation experiments, however, indicate only physical association between the partners. To explore whether these transporters functionally interact, we co-expressed the wild type and inactive mutant variants of ABCG1 and ABCG4 in various combinations in HEK293 cells, and assessed the apoptosis as a functional readout. As demonstrated in Fig 6, co-expression of ABCG4_K108M_ with wt ABCG1 markedly reduced the fraction of apoptotic cells as compared to the cultures transfected with wt ABCG1 alone, suggesting a functional interaction between ABCG1 and ABCG4. ABCG1_K124M_ also diminished ABCG1-induced apoptosis, but the corresponding inactive mutant form of ABCG2 (ABCG2_K86M_) had no effect. To exclude the possibility that the decreased apoptosis in the co-transfected cultures is due to the reduced expression level of the wt transporter, we performed a Western blot analysis with single and co-transfected cultures. The expression of ABCG1 remained unaltered when co-expressed with ABCG4_K108M_ or ABCG2_K86M_ (S4 Fig).

Similar results were obtained with an inverse experimental arrangement, when the wt ABCG4 was co-expressed with ABCG1_K124M_, ABCG4_K108M_, or ABCG2_K86M_. ABCG4-induced apoptosis was diminished by the inactive form of ABCG1 or ABCG4 but not with that of ABCG2 (Fig 6A). These observations are in accordance with results of the co-immunoprecipitation experiments, demonstrating that ABCG4 forms functional homodimers (Fig 1B) as well as heterodimers with ABCG1 (Fig 2A), but not with ABCG2 (Fig 2C). To verify the co-expression of the two proteins in the co-transfected cultures, we performed dual immunostaining, whenever isotype differences of the specific antibodies allowed. These experiments demonstrated that both transfected proteins were expressed and exhibited largely overlapping expression patterns in these cultures (Fig 6B).

The effect of the inactive mutant variants on the apoptosis induced by ABCG1 or ABCG4 was quantitatively evaluated (Fig 6C). This analysis confirmed the results of the representative experiments shown in Fig 6A. It is important to mention that in these co-transfection experiments the ratio of applied DNA constructs of wile type vs. inactive mutant form was 1:2. This biased co-expression helped to form more wt-KM dimers and less wt-wt homodimer, enhancing the potential inhibitory effect of the inactive mutant form.

Furthermore, the short isoform of ABCG1 was also included in this set of experiments. We found that the inactive mutant form of ABCG1S reduced the apoptosis induced by the long isoform, and vice versa, indicating that these isoforms can functionally interact with one another. Moreover, ABCG4 can form functional heterodimers with both ABCG1 isoforms, since ABCG4_K108M_ diminished apoptosis induced by either ABCG1 isoform. Similar to ABCG4 and the long isoform of ABCG1, the short ABCG1 variant does not seem to interact with ABCG2, since ABCG2_K86M_ had no effect on the ABCG1S-induced apoptosis.

Despite the fact that there was no obvious difference in the transfection efficacy and expression levels of ABCG4 and ABCG1, the former protein resulted in three-fold less apoptotic cells in the cell cultures. The short variant of ABCG1, however, induced apoptosis to an extent similar to that was observed in cultures transfected with the long ABCG1 isoform, suggesting that the 12 amino acid-long insert is not required for this function.

## Discussion

Although ABCG1 and ABCG4 are closely related to ABCG2, which is a widely studied, well-characterized ABC transporter, numerous basic issues about ABCG1, and especially ABCG4, are still unclear or disputed. Our present study focuses on some fundamental characteristics of ABCG4, addressing several open questions about this enigmatic ABC protein.

One of these unresolved issues is the subcellular localization of ABCG4. Most mammalian ABC transporters reside in the plasma membrane, and mediate the transport of substances from the cell interior to the external space. However, several ABC transporters are known to localize and function in intracellular compartments. An evident example for this is the ER-resident TAP1/TAP2 transporter dimer, and members of the ABCD subfamily are also intracellularly functioning transporters. In addition, the localization of some ABC transporter, e.g. the ABCB6 half-transporter is heavily disputed [29, 30]. Overexpression of plasma membrane resident proteins may also lead to their accumulation in unconventional cellular locations. For example, ABCC1/MRP1 was shown to sequestrate drugs in intracellular compartments, when overexpressed [31]. By all means, membrane transporter proteins travel through several cellular compartments during their life span, even though their business end is not necessarily in those organelles. As an example, ABCB11/BSEP continuously cycles between the apical surface and recycling endosomes in the hepatocytes [32]. Moreover, a major fraction of this transporter, which is commonly thought to be a plasma membrane-resident protein, is localized to the intracellular endosomal pool [33]. It has been reported for several other ABC proteins, including ABCC2, ABCG1, and ABCG2, that a fraction of transporter is localized to recycling endosomes [34–36].

Most members of the ABCG subfamily, such as ABCG2 and ABCG5/ABCG8, are thought to function in the plasma membrane, whereas the cellular target of ABCG1 is controversial. Our previous study on ABCG1 demonstrated predominant plasma membrane localization [24], which is in agreement with the results of numerous other groups [7, 10, 20, 37–39]. Contrary, several studies reported that ABCG1 localizes intracellularly and proposed a functional role for ABCG1 in the endosomal system [9, 16, 35, 40, 41]. However, it has recently been reported that a mutant form of the ABCG1 was used in one of these studies, and the substitution of Leu to Pro at position 550 and 562 in the murine and human ABCG1, respectively, alters its plasma membrane localization, and the mutant form localizes intracellularly [42]. Interchange between the intracellular ABCG1 pool and the cell surface has also been suggested [8, 23], although it has been questioned by others [39].

The subcellular localization of ABCG4 is also disputed. Tarr et al., when expressing epitope-tagged ABCG4 in primary neurons, astrocytes and Cos-7 cells, showed endosomal localization [16], whereas others demonstrated plasma membrane localization of ABCG4 [7, 38]. It should be noted that tagging may significantly affect trafficking, and thereby the cellular localization of a membrane protein. Most studies reporting intracellularly localized ABCG1 also used epitope-tagged versions of the transporter [8, 9, 16, 40, 41]. Endogenous expression of ABCG4 was demonstrated in megakaryocyte progenitors, where ABCG4 was found predominantly in the Golgi and trans-Golgi compartments [26]. Although the results have been interpreted differently, taking a closer look at the subcellular localization experiments of this study, a substantial fraction of ABCG4 can be observed in the endosomal system and even in the plasma membrane.

In the present work, we analyzed the cellular distribution of untagged ABCG4 by using a highly specific antibody, which was developed in our laboratory. Predominant plasma membrane localization was observed not only in transfected HEK293 cells, but also in human (SH-SY5Y) and mouse (N2a) neuronal cells. A smaller fraction of ABCG4 was found in the Golgi apparatus, which is not surprising for a membrane protein processed in the Golgi. It is plausible that the accumulation of ABCG4 in the Golgi complex is due to the overexpression of the protein. However, similar level of overexpression of the inactive mutant variant resulted in selective plasma membrane localization. Considering that the active ABCG4 triggers apoptosis, the expression seen in the Golgi complex can also be a consequence of the morphological changes concomitant with apoptosis. Interestingly, as mentioned earlier, endogenous expression of ABCG4 was also seen in the Golgi and trans-Golgi compartments in megakaryocyte progenitor cells [26]. In addition to that seen in the plasma membrane and in the Golgi complex, a small fraction of ABCG4 was found in small punctate structures, which do not co-localize with any of the used markers. These puncta can be either post-Golgi vesicles or recycling endosomes delivering ABCG4 to the cell surface; or alternatively endocytic vesicles, which transfer the excess of the protein to the degradation pathway. To explore the course or the character of these vesicles, further studies involving dynamic experimental approaches are needed. Using untagged protein and a different approach, our study also verified the finding of Engel et al., who demonstrated that ABCG4 is likely not glycosylated [20].

Another focus of our study was the dimerization properties of ABCG4 and ABCG1 proteins. Co-immunoprecipitation experiments demonstrated a physical interaction between the GFP- and non-tagged versions of ABCG4, implying homodimer formation. The ratio of precipitated proteins was approximately 1:1 (Fig 1B), which suggests dimer formation rather than oligomerization. Since we applied the untagged version in a great excess to promote the interaction, this bias would be reflected at higher degree oligomerization. For instance, 1:3 tagged-untagged ratio is expected in the case of tetramer formation, etc., which was not observed here. Similar results were obtained with ABCG1 (Fig 1C), which verifies the homodimerization of ABCG1 reported in previous studies [8, 10].

In addition to a physical interaction, we exploited the apoptotic effect of these ABC half transporters as a functional readout. The inactive mutant variant of ABCG4 diminished the apoptosis induced by the wild type ABCG4, and similarly the inactive ABCG1 blocked the ABCG1-stimulated apoptosis. These results demonstrate not just a physical interaction, but the intimate functional cooperativity between the two halves of the dimers. In addition, we demonstrated that the short isoform of ABCG1, which is the predominant version in humans, also forms homodimers. Moreover, this protein is capable of dimerizing with the full-length ABCG1.

It is plausible to assume that ABCG1 and ABCG4 can also form heterodimers. They share 72% amino acid identity, and have partially overlapping tissue distribution [15, 16, 18]. Closely related ABC half transporters, such as ABCG5 and ABCG8 are obligate heterodimers [6], and the *Drosophila* orthologues of ABCG1 are known to form heterodimers. Both co-immunoprecipitation and the functional assays confirmed the heterodimer formation between ABCG4 and either isoform of ABCG1. Inhibitory effect of the inactive half transporters (ABCG4_K108M_, as well as the long and short forms of ABCG1_K124M_) on ABCG4-induced apoptosis also demonstrates that the function leading to apoptosis requires both nucleotide-binding domains to be active. In a previous study using epitope-tagged variants, detectable amounts of ABCG1 and ABCG4 were seen in the immunoprecipitates pulled down with GFP-tagged ABCG2 [20]. Despite this, the authors do not support the idea of heterodimerization of ABCG2 with ABCG1 or ABCG4. Our study demonstrates that no interaction with ABCG2 is formed with either ABCG4 or ABCG1, since no ABCG2 was detected in the immunoprecipitated samples even with a Western blot analysis using an extreme long exposition time (data not shown). The advantage of our system over the previous study, that no tagging was applied in our study.

A previous study by Sano et al. demonstrated that ABCG1 and ABCG4 are localized to two distinct plasma membrane domains. ABCG1 was found in membrane rafts resistant to both Triton X-100 and Brij 96, whereas ABCG4 resides in rafts resistant to Brij 96 only and solubilized by Triton X-100 [38]. Moreover, ABCG1 co-localize with flotillin-1, while ABCG4 does not. Whether the ABCG1/ABCG4 heterodimer prefers the Triton X-100-resistant raft or localized to the same membrane domain as the ABCG4 homodimer, or to both, remains an open question. It is also unknown whether interchange between these different membrane domains takes place. The most plausible model for heterodimer formation is that the monomers are assembled in the endoplasmic reticulum and delivered together to the cell surface; however, there is no data available that exclude the possibility of reassembly of ABC half transporter dimers in the target compartment.

Heterodimer formation is frequently concomitant with similar transcriptional regulation. For instance, the *ABCG5* and *ABCG8* possess a common promoter, which transcription is induced by LXR [43, 44]. The expression of both *ABCG1* and *ABCG4* genes were also reported to be upregulated in response to LXR and RXR agonists in several studies [13, 14, 17, 21, 24, 25, 45]. However, in certain cell types, such as oligodendrocytes and placental endothelial cells, only ABCG1 but not ABCG4 was induced by LXR agonist [46, 47]. Previous studies suggested that ABCG1 and ABCG4 can be co-expressed in the central nervous system and the eye [15, 16, 18], however, these studies have not directly shown interactions between these proteins. Our study clearly demonstrate both physical and functional interactions between ABCG4 and ABCG1, suggesting that heterodimer formation may occur in tissues, where both proteins are expressed.

Similarly, to ABCG1, ABCG4 has been proposed to participate in lipid metabolism, since the mRNA expression level of ABCG4 was induced by LXR agonists in several cell types, including human primary macrophages, Thp-1 cells, megakaryocyte progenitors, and neuronal cells [13, 14, 17, 25, 26]. However, no induction by LXR was found in certain cell types, including astrocytes, placental endothelial cells, and oligodendrocytes [15, 46, 47]. Heterologous expression of mouse ABCG4 in HEK cells resulted in increased HDL-dependent cholesterol efflux [14]. In addition, the level of certain sterols was found to be elevated in the brain of ABCG4-depleted mice [15, 18]. Despite these findings, the role of ABCG4 in sterol/lipid metabolism remains unclear. Cholesterol efflux from macrophages of wild type and ABCG4 knock-out mice was identical, and LXR-induced cholesterol efflux was independent of the presence of ABCG4 [23]. A more recent study suggested a protective role for ABCG4 in the defense against thrombosis and atherosclerosis [26]. In the proposed model, ABCG4, residing in the Golgi and trans-Golgi compartments of megakaryocyte progenitor cells, promotes cholesterol transfer to HDL. Decrease in the membrane cholesterol content is sensed by the LYN kinase, activating E3 ligase c-CBL, which results in the down-regulation of the thrombopoietin receptor c-MPL. This series of events ultimately leads to the suppression of the megakaryocyte progenitor proliferation and diminishing of platelet production [26]. This model is in line with the long-established idea of the intimate relationship between cholesterol metabolism and proliferative responses.

Our observations suggest an alternative role for ABCG4. The altered cell morphology, reduced cell growth capacity, high number of Annexin V positive cells, and elevated caspase-3 activity together demonstrate that the expression of ABCG4 is associated with apoptotic cell death. Similar apoptotic effect induced by the long isoform of ABCG1 has previously been reported by our group [24]. Here we demonstrate the first time that the short isoform of ABCG1, which is the predominant form in humans, also induces apoptosis. The apoptotic effects of these ABC half transporters are clearly connected with their function, since i) the catalytic site mutant variants (ABCG4_K108M_ and ABCG1_K124M_) do not elicit apoptosis, and ii) co-expression with the inactive mutants attenuates the apoptosis induced by the wild type forms. Although about three-fold more apoptotic cells was observed in ABCG1-expressing cells than in the ABCG4-transfected cultures (13.6±0.91% vs. 4.45±0.38%), only a slight difference is seen in the growing curves shown in Fig 4A. With the given numbers, the ratio of non-apoptotic cells in the two cultures is about 0.9, therefore, only a small difference in the growth kinetics is expected despite the substantial difference in the number of apoptotic cells.

How the functional expression of these ABCG proteins lead to programmed cell death, remains elusive. One possible explanation is that overexpression of these transporters put an energy burden on the cells, and ATP depletion results ultimately in apoptosis. However, the basal ATPase activity of ABCG4 determined in Sf9 membranes is much smaller than that of ABCG2 [19], and the overexpression of the latter protein did not lead to apoptosis (Fig 5B). Thus, this explanation seems unlikely. Nevertheless, a connection between activation of LXRα and apoptosis induction has been previously reported. Neuroblastoma cells treated with LXR agonists showed signs of apoptosis, as well as increase in both pro-apoptotic gene expressions and reactive oxygen species (ROS) production [48]. Thus, a plausible explanation for our observation is that these LXR-inducible gene products, ABCG1 and ABCG4, are associated with ROS production, which results in cell damage. However, no detectable elevation in ROS levels in ABCG1- and ABCG4-transfected cells was observed in our hands (data not shown).

The apoptotic effect of ABCG4 and ABCG1 shown in our experiments seemingly contradicts previous observations suggesting a role for these proteins in cellular efflux of lipids and sterols. However, these two phenomena can be reconciled if we consider that deprivation of lipid/sterol levels of cells distracts cellular homeostasis, which leads to programmed cell death [49, 50]. This is also in line with the previously mentioned observation that induction with LXR agonists is concomitant with elevation in the expression of pro-apoptotic genes [48]. Considering that ABCG4 and ABCG1 are localized to the plasma membrane, and potentially involved in lipid and sterol transport, they may change local microenvironment of the membrane, causing alterations in the distribution of adhesion molecules and/or assembly of adhesion complexes. This may ultimately lead to detachment-induced programmed cell death, which has been termed anoikis [51, 52].

To explore the question, whether the apoptotic effects of ABCG4 and ABCG1 observed in *in vitro* systems has a physiological relevance, the expression pattern of these transporters ought to be taken into account. In general, both transporters are expressed in macrophage-type cells. ABCG1 is present in monocyte-derived macrophages and Kupffer cells, liver-specific macrophages, whereas ABCG4 is expressed in certain cells of the central nervous system, e.g., microglial cells. In addition, ABCG4 was found in megakaryocyte progenitor cells in the bone marrow [26]. Normally the clearance of apoptotic bodies is very fast and the proteins are efficiently cleaved by proteases during apoptosis, thus it is hard to detect the remnants *in vivo*. However, reports proposed a role for ABCG4 in Alzheimer’s disease (AD), demonstrating increased expression of ABCG4 in microglial-like cells in the brain of AD patients [53]. In addition, ABCG4-mediated efflux of amyloid-β peptide (Aβ) and expression of ABCG4 in the brain capillaries were also shown [54]. These results along with our observations may suggest a role for ABCG4 in a clearance system for the elimination of Aβ peptide and cell debris, which is an important mechanism to hinder AD development. Most recently, the role of microglial-driven clearance and microglial apoptosis in AD pathogenesis attracted much attention [55, 56]. Moreover, it has been proposed that elimination of cell debris by microglial cells may contribute to the prevention of multiple sclerosis and other neurological disorders.

The function of ABCG4 in megakaryocyte progenitors has been connected to proliferative responses in a negative regulatory fashion [26]. ABCG4 deficiency resulted in increased cell number, and elevated platelet production. Our finding, linking ABCG4 function to apoptosis, is in line with these observations. Apoptosis is an essential mechanism in the hematopoiesis, and especially important in development of myeloid cells (reviewed in [57]). In addition to its proliferative effect, thrombopoietin was shown to inhibit apoptosis of multipotent hematopoietic progenitor cells, megakaryocyte progenitors, and immature megakaryocytes [58–60]. ABCG4 has been reported to reduce thrombopoietin response in the megakaryocyte progenitors [26]. Whether ABCG4-associated apoptosis has a role in the regulation of megakaryocyte maturation, is an intriguing issue, which requires further studies to be answered.

In summary, we demonstrated that ABCG4 can function either as a homodimer or as heterodimer with either long or short isoforms of ABCG1. ABCG4 is predominantly localized to the plasma membrane, and induces apoptosis in various cells. The apoptotic morphology of ABCG1-expressing cells was observed by others [37], although no detailed analysis were carried out to explore this phenomenon prior to our study. The mechanism how ABCG4 (or ABCG1) function leads to programmed cell death remains unclear. However, the uncovered phenomenon, the ABCG4-induced apoptosis, brings up a novel area to be explored, which may assist in better understanding of dysfunctional clearance mechanisms in certain neurological disorders.

## Supporting Information

S1 Fig Co-immunoprecipitation of ABCG1 and ABCG4 proteins using anti-ABCG1 antibody. HEK293 cells were transfected with ABCG4 (to minimize apoptosis in the cultures, the inactive mutant form, G4KM was used), or co-transfected with G4KM and wt ABCG1 (G1) or its inactive variant (G1KM). 24 hours after transfection, the cells were lysed and immunoprecipitated with anti-ABCG1 (αG1) antibody. The heterodimer formation was investigated by using anti-ABCG4 (αG4) antibody for development of the Western blots of the precipitates (lower panels). The protein expressions of G4KM and the ABCG1 variants were verified by Western analysis of the cell lysates (input). These results are consistent with the inverse experiments shown in Fig 1, demonstrating heterodimer formation between ABCG4 and ABCG1 variants.
(TIF)

S2 Fig Parallel detection of apoptotic cells and ABCG4 expression. HEK293 cells were transfected with ABCG4, and the fraction of apoptotic cells in the culture was determined by Annexin V binding at the indicated time points following transfection (A). In parallel, the expression of ABCG4 was assessed by Western blot analysis (B). The relative protein expression was determined by densitometry using the Na^+^K^+^ ATPase for loading control (C). The dose-effect curve demonstrates close correlation between ABCG4 expression and apoptosis (D).
(TIF)

S3 Fig Apoptosis induced by both isoforms of ABCG1. HEK293 cells were transfected with the full-length (G1) or short isoform (G1S) of ABCG1, or with their inactive mutant variants (G1KM or G1SKM). Apoptotic cells in cultures were visualized by fluorescently labeled Annexin V (green). Lower panels depict nuclear staining of the same cell cultures using Hoechst 33342 dye.
(TIF)

S4 Fig Expression levels of wt ABCG1 in single- or co-transfected cultures. HEK cells were transfected with the wt ABCG1 alone, or co-transfected with the inactive mutant variant of ABCG1 (G1KM), ABCG4 (G4KM), ABCG1S (G1SKM), or ABCG2 (G2KM). Western blots demonstrate that the expression level of the wt protein is not altered by the presence of the inactive forms. Elevation in the total expression level of ABCG1 was only observed when the wild type and the inactive mutant forms of ABCG1 were co-expressed. For loading control the -Na^+^K^+^ ATPase was used.
(TIF)
