# Supplementary figures and images for: Functional Cooperativity between ABCG4 and ABCG1 Isoforms
Source: PLoS One. 2016 May 26;11(5):e0156516. doi: 10.1371/journal.pone.0156516 (PMC4882005; doi:10.1371/journal.pone.0156516)

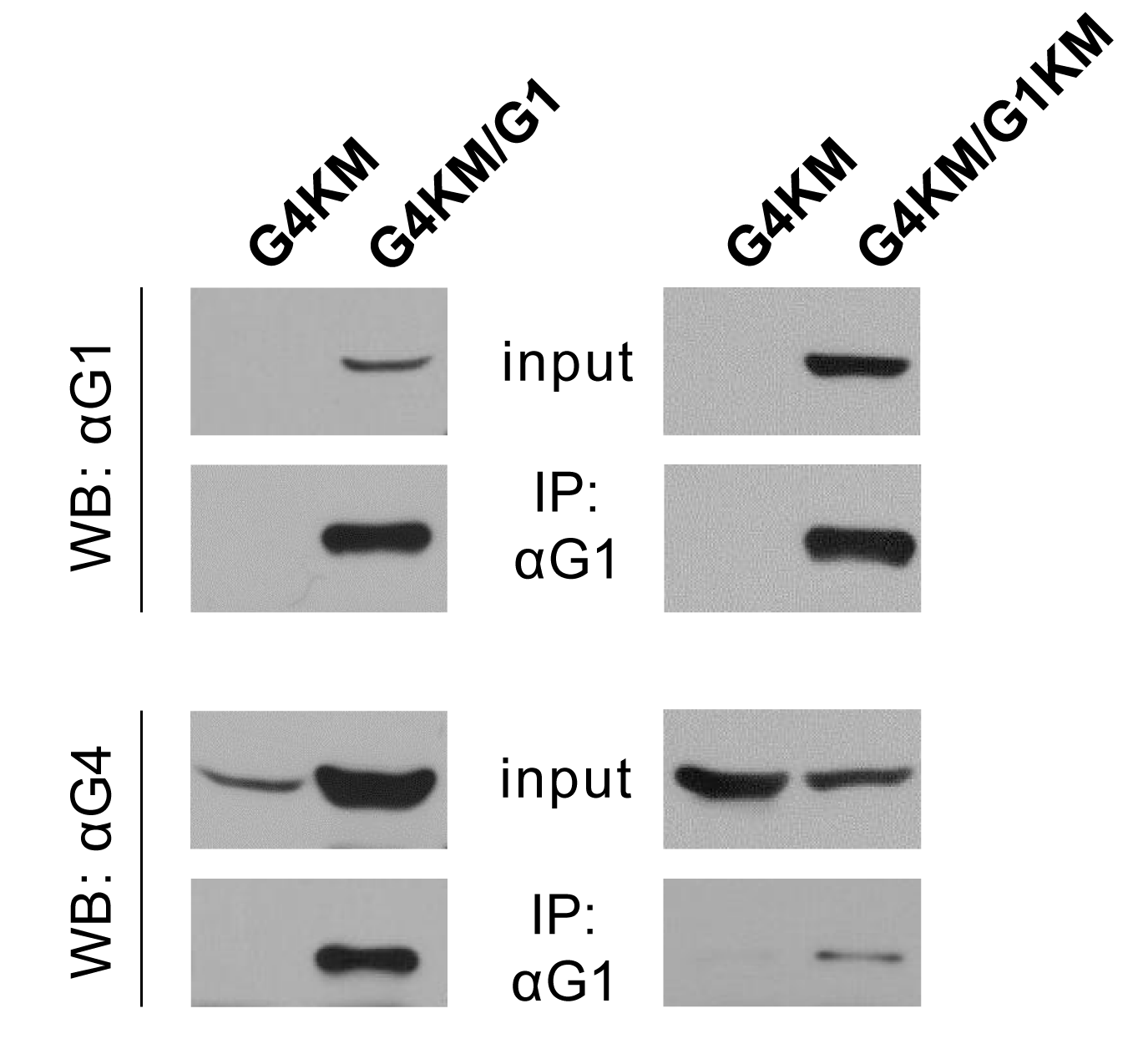

Supplement: S1 Fig — HEK293 cells were transfected with ABCG4 (to minimize apoptosis in the cultures, the inactive mutant form, G4KM was used), or co-transfected with G4KM and wt ABCG1 (G1) or its inactive variant (G1KM). 24 hours after transfection, the cells were lysed and immunoprecipitated with anti-ABCG1 (αG1) antibody. The heterodimer formation was investigated by using anti-ABCG4 (αG4) antibody for development of the Western blots of the precipitates (lower panels). The protein expressions of G4KM and the ABCG1 variants were verified by Western analysis of the cell lysates (input). These results are consistent with the inverse experiments shown in Fig 1, demonstrating heterodimer formation between ABCG4 and ABCG1 variants. (TIF) [file pone.0156516.s001.tif]

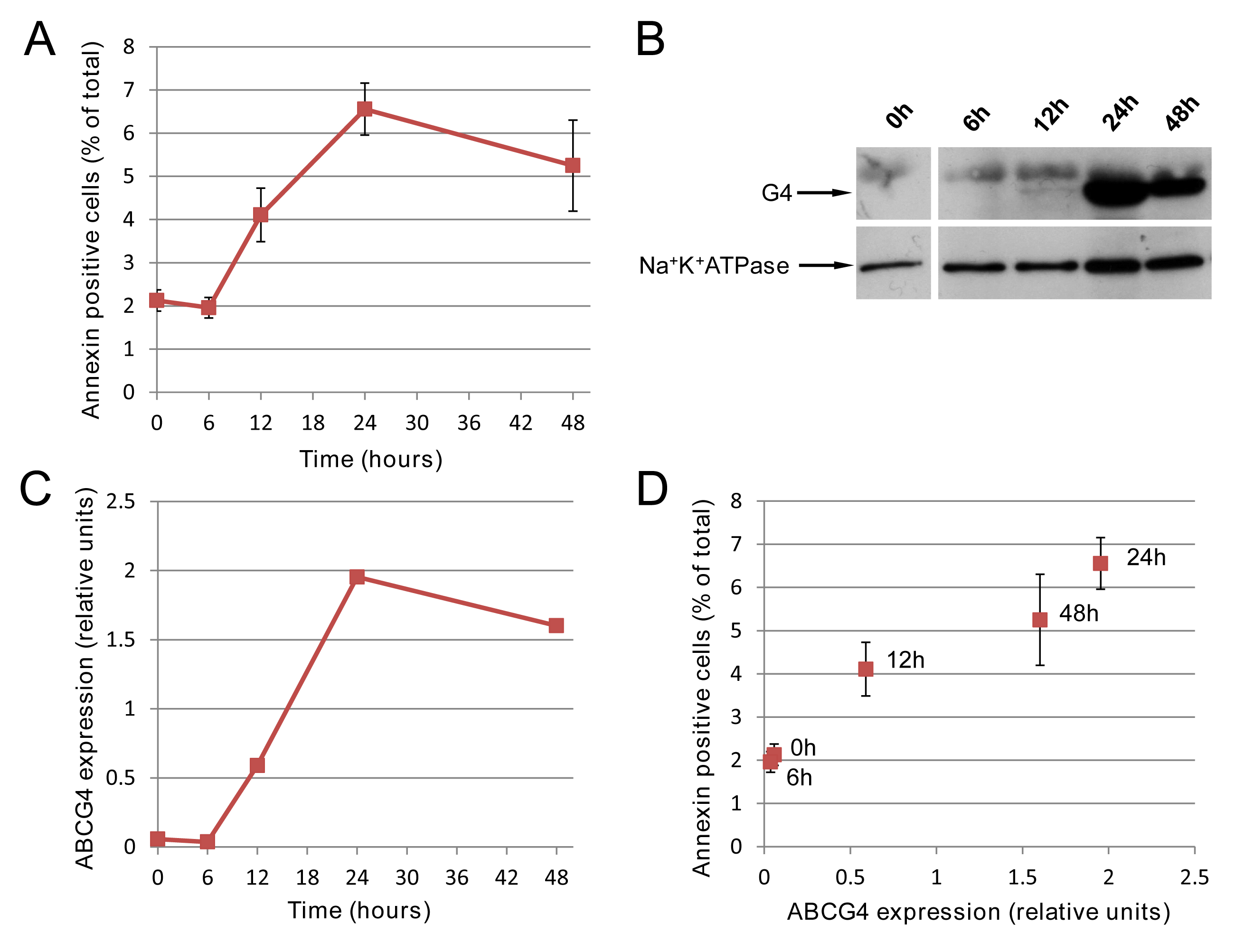

Supplement: S2 Fig — HEK293 cells were transfected with ABCG4, and the fraction of apoptotic cells in the culture was determined by Annexin V binding at the indicated time points following transfection (A). In parallel, the expression of ABCG4 was assessed by Western blot analysis (B). The relative protein expression was determined by densitometry using the Na+K+ ATPase for loading control (C). The dose-effect curve demonstrates close correlation between ABCG4 expression and apoptosis (D). (TIF) [file pone.0156516.s002.tif]

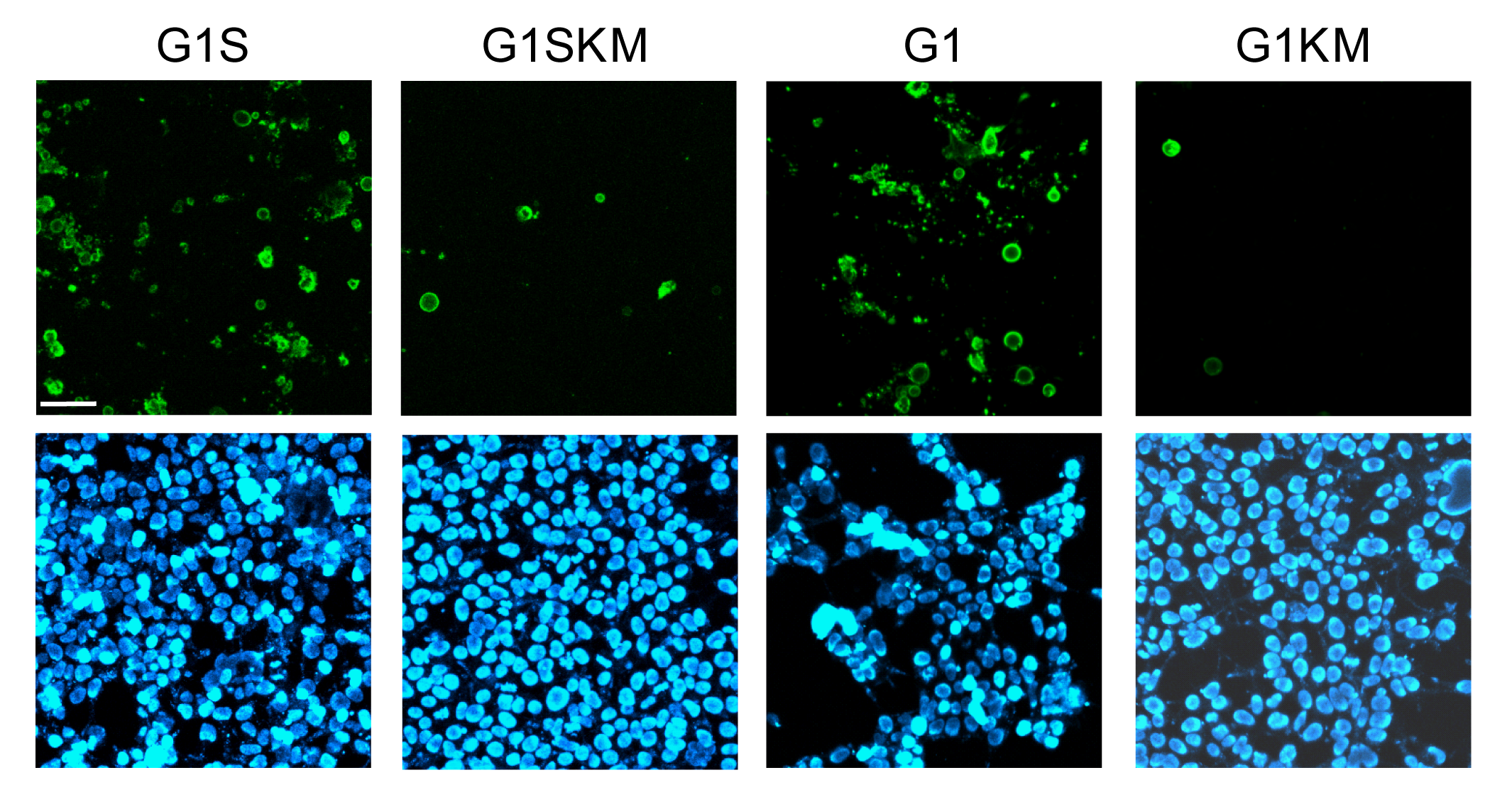

Supplement: S3 Fig — HEK293 cells were transfected with the full-length (G1) or short isoform (G1S) of ABCG1, or with their inactive mutant variants (G1KM or G1SKM). Apoptotic cells in cultures were visualized by fluorescently labeled Annexin V (green). Lower panels depict nuclear staining of the same cell cultures using Hoechst 33342 dye. (TIF) [file pone.0156516.s003.tif]

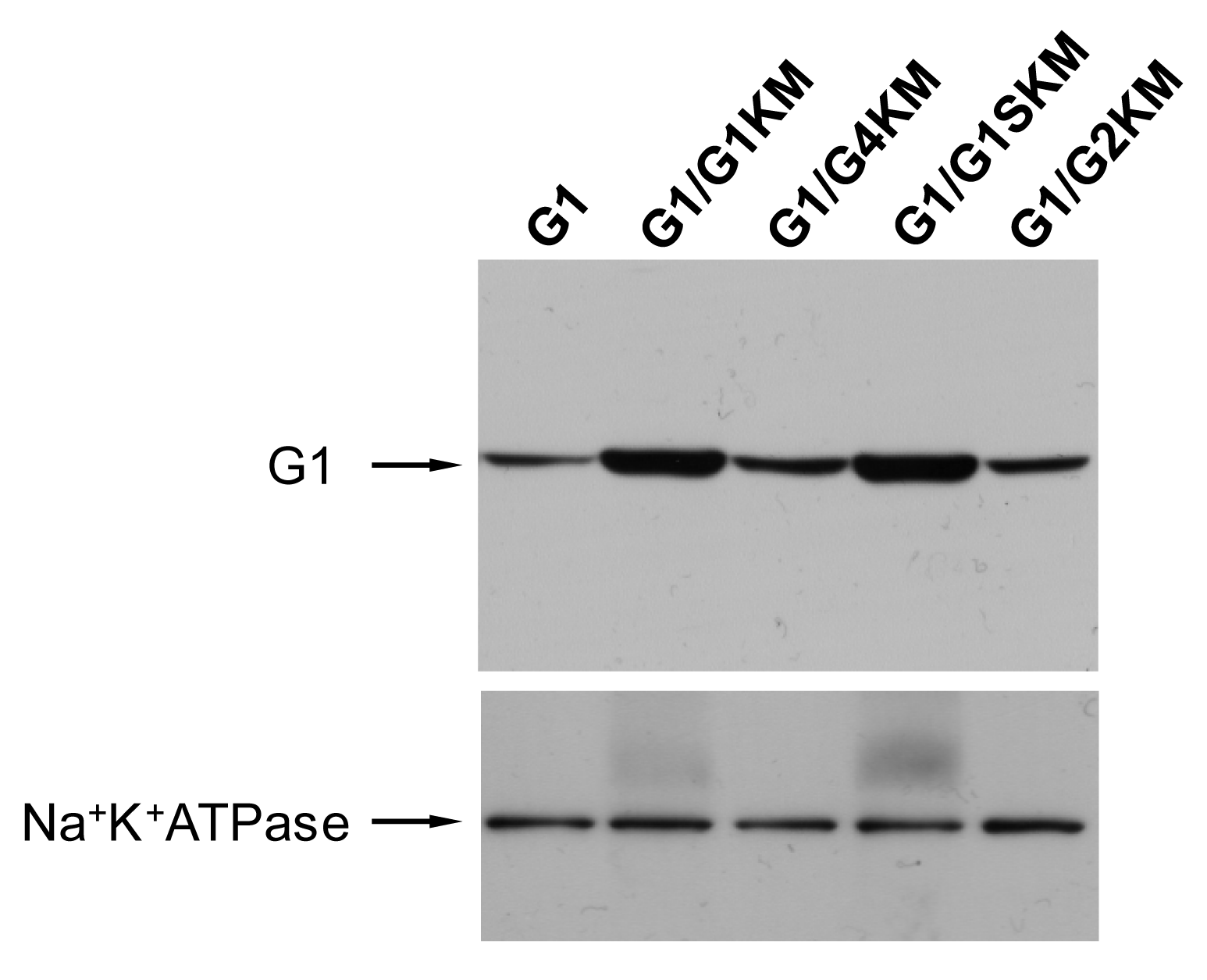

Supplement: S4 Fig — HEK cells were transfected with the wt ABCG1 alone, or co-transfected with the inactive mutant variant of ABCG1 (G1KM), ABCG4 (G4KM), ABCG1S (G1SKM), or ABCG2 (G2KM). Western blots demonstrate that the expression level of the wt protein is not altered by the presence of the inactive forms. Elevation in the total expression level of ABCG1 was only observed when the wild type and the inactive mutant forms of ABCG1 were co-expressed. For loading control the -Na+K+ ATPase was used. (TIF) [file pone.0156516.s004.tif]
